# A case report of unexplained jejunal telangiectasia complicated with bleeding

**DOI:** 10.1016/j.ijscr.2020.01.062

**Published:** 2020-02-06

**Authors:** Wen-jun Zhang, You-shan Huang, Zheng-ming Zhu, Hong-liang Luo

**Affiliations:** The Second Affiliated Hospital, Nanchang University, Nanchang City, Jiangxi Province, 343000, China

**Keywords:** Small intestinal telangiectasia, Jejunum, Bleeding, Treatment

## Abstract

**Introduction:**

Small intestinal telangiectasia is a clinically rare disease, which is mainly characterized by gastrointestinal bleeding. There is a lack of specific and effective diagnostic methods in clinical practice due to its unknown etiology and difficult localization, it is often difficult to find the location of the lesion even through observation, touch, endoscope or intestinal incision.

**Case presentation:**

A 39-year-old female patient, who had black stool twice without obvious inducement 3 days ago, and came to our hospital for treatment and was hospitalized with gastrointestinal bleeding. the main manifestation of this patient was repeated black stool, sometimes dark red. Initially, we considered other diseases of the digestive tract (gastric ulcers, duodenal ulcers and intestinal tumors). However, no abnormalities were found by CT, gastroscopy and enteroscopy. Later, we considered that there was a greater possibility of intestinal vascular disease, and then blood clots was found in the upper and middle segment of the jejunum through capsule endoscopy, but no bleeding site was found. Therefore, we decided to open the abdomen for further intraoperative enteroscopy exploration, finally found the bleeding point, and then stopped the bleeding by suture. Later, through further follow-up, no rebleeding was found in the patient.

**Conclusion:**

Jejunal telangiectasia with bleeding is a very rare intestinal vascular disease, which is difficult to identify and diagnose clinically. Therefore, the possibility of this disease should be considered in patients with negative results through some examinations such as gastroscopy, enteroscopy, gastrointestinal barium meal radiography, etc. In order to treat in time and prevent bleeding

## Highlights

• Jejunal telangiectasia with bleeding is relatively rare in clinic.
• The main clinical manifestation of small intestinal telangiectasia is hematochezia.
• This disease is prone to recurrent bleeding.
• There is a lack of specific and effective diagnostic methods in clinical.
• We gave some experience and suggestions in the process of surgical treatment.

## 1 Introduction

Small intestinal telangiectasia is a relatively rare vascular disease of the digestive tract, which occurs mainly in the right half of the colon or cecum, and rarely in the small intestine. The main manifestations are hematochezia, black stool, fecal occult blood and anemia, without any prodromal symptoms and specific signs [1,2]. Clinically, it is difficult to locate and diagnose even through gastroscopy, enteroscope, barium meal angiography and other inspection methods, it is difficult to distinguish it from other diseases, resulting in lack of pertinence in diagnosis and treatment, and easy to misdiagnose and delay treatment [3,4]. Therefore, the possibility of occurrence of this disease should be considered in patients with recurrent gastrointestinal bleeding who can not find the cause after corresponding examination. In this report, we reviewed a patient with unexplained jejunal telangiectasia complicated with bleeding, the main manifestation of this patient was repeated black stool, sometimes dark red. Initially, we considered other diseases of the digestive tract (such as gastric ulcers, duodenal ulcers and intestinal tumors), and no abnormalities were found by CT, gastroscopy and enteroscopy. Later, we considered that there was a greater possibility of intestinal vascular disease, and then blood clots was found in the upper and middle segment of the jejunum through capsule endoscopy, but no bleeding site was found. Therefore, we decided to open the abdomen for further intraoperative enteroscopy exploration, and finally found the bleeding point. Through this case report, our main purpose is to provide a valuable reference and advice for clinicians in the diagnosis and treatment of this disease.

## 2 Case representation

**Fig. 1:**
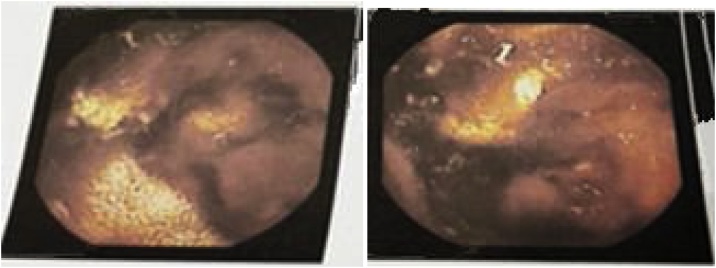
Schematic diagram of the results of capsule endoscopy. The photography by swallowing capsule endoscopic. The time for capsule endoscope to pass through the small intestine was 3 h and 34 min. When the capsule endoscope was swallowed for 48 min, a large amount of blood clots could be seen in the jejunum and the entire visual field was covered by the blood clots, which showed unclear and no bleeding points were found.

A 39-year-old female patient, who had black stool twice without obvious inducement 3 days ago, accompanied by dizziness, and no other symptoms such as abdominal distension, abdominal pain, cold, and fever. After symptomatic treatment in the local hospital, the condition did not improve, so came to our hospital for further treatment and was hospitalized with gastrointestinal bleeding. Physical examination: T 36.7 °C, P 78/min, R 20/min, BP 99/62 mmHg, moderate anemia (Hb 85 g/L), no other abnormal signs and previous history of other diseases. After admission, through the preliminary examination of abdominal CT scanning, the results showed that hepatic cysts, small calcifications in the left lobe of the liver, and gallbladder inflammation, but no abnormalities in the intestinal tract. Subsequently, through electronic gastroscopy and enteroscopy, the results of the gastroscopy showed that patchy erythema could be seen in the gastric antrum, while in other parts of the stomach, the mucosa was smooth, the folds were intact, and the gastric peristalsis was good, which was diagnosed as superficial gastritis. The enteroscopy showed that, when the electronic enteroscope was inserted into the end of the ileum, a small amount of brown liquid could be seen, the intestinal mucosa was smooth, and the ileocecal valve is good. While a lot of coffee-like liquid could be seen in the colon cavity, but no bleeding site was found. At this time, we discussed and evaluated the relevant research results and case data, and highly suspected the possibility of intestinal vascular disease. In order to make a clear diagnosis and locate the bleeding site, further capsule endoscopy was performed (the whole photography time of capsule endoscopy was 11 h and 30 min). After the capsule endoscopy was swallowed, it was sent to the duodenum under the gastroscope, and entered the colon in 3 h and 27 min. Fortunately, some gains were obtained, at 48 min, there were a lot of fluid and blood clots in the upper and middle segment of the jejunum could be seen, but the entire field of vision was poorly blurred and no bleeding point was still found (Fig. 1). Through the results of capsule endoscopy, we were full of confidence in the previous judgment, and initially positioned the bleeding site in the jejunum.

**Fig. 2:**
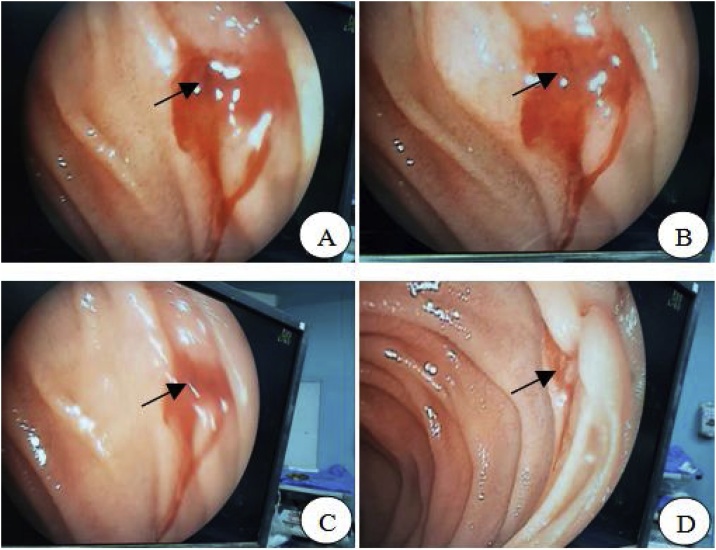
Intraoperative diagram of jejunal telangiectasia with bleeding. The site of jejunal telangiectasia with bleeding. A, B and C: bright red blood flowing out of the jejunum about 10 cm from the ligament of Traitz (the black arrow indicates the location of the bleeding point) and dilated blood vessels could be seen during operation. The mucosa of the intestinal lumen in other parts was smooth and no abnormalities were found. D: After the bleeding site was sutured, no blood outflow was seen at the bleeding point, and the purpose of hemostasis was achieved.

After a week of treatment through nutritional support, rehydration and correction of anemia, the patient still had recurrent and intermittent black stool, and her condition was not very stable. Therefore, we decided to explore by laparotomy [5]. Based on the results of capsule endoscopy, the dark black part of the jejunum was found, and then the jejunum was cut at about 5 cm above it for intraoperative enteroscopy. The bleeding spot was finally found (located at about 10 cm from jejunum to the ligament of Traitz) and then the hemostasis was stopped by suture. Finally, it was diagnosed as jejunal telangiectasia complicated with bleeding (Fig. 2). After hemostasis during the operation, a thorough and careful exploration of other intestines was performed, and no other bleeding sites were found. After operation, the patient's condition was stable and the symptoms of hematochezia and anemia were controlled through symptomatic treatment.

## 3 Discussion

Sir William Osler et al., the first proposed the disease of telangiectasia in 1901, and then first reported by Sutton et al. in 1964 [6]. The main manifestation of telangiectasia were recurrent epistaxis and mucosal telangiectasia, and arteriovenous malformations may occur in other organs (such as brain, spinal cord, liver, gastrointestinal tract, etc.) [[7], [8], [9]]. However, the incidence of small intestinal telangiectasia is low, which is more common in the elderly, probable cause is the weakened elasticity of the vascular wall and the obstruction of intramucosal venous reflux, resulting in telangiectasia and mucosal ischemia. The main symptom of this disease is recurrent gastrointestinal bleeding, which is more common sign as “tarry stools”. Thus, it is difficult to diagnose and treat clinically due to its diverse symptoms and signs [10]. Traditional non-invasive examination methods (such as barium meal radiography of small intestine) are difficult to detect and diagnose small intestinal telangiectasia with bleeding, which is easy to misdiagnose [11]. For example, during small bowel endoscopy, experienced operators can observe more than 90% of the small intestinal mucosa, and can perform electrocautery to stop bleeding caused by arteriovenous malformations. However, the examination of small bowel endoscopy is relatively difficult, the requirement for the intestinal tract is high, and the diagnosis rate of bleeding is not high [12]. Compared with intestinal endoscopy, capsule endoscopy is a direct imaging method, improves the detection rate and diagnosis rate of small intestinal telangiectasia with bleeding. However, there are some drawbacks to capsule endoscopy, such as, inflation, irrigation, repeated local observation, biopsy and treatment can not be performed like the conventional endoscopy, and the examination time is long. For patients with obvious active bleeding and intestinal hemorrhage, the visual field of capsule endoscope is not clear and easy to miss diagnosis [13,14]. Radionuclide scanning has high sensitivity in the diagnosis of hemorrhage, but the localization value is low, which can cause false positive results by the accumulation of blood in the intestinal cavity, and the examination results are often negative during the intermission period of bleeding [15]. Mesenteric angiography is of high value in the diagnosis of small intestinal telangiectasia with bleeding. When the bleeding volume reaches 0.5 ml/min, it can show the overflow of contrast medium and the condition of blood vessels (such as vascular distortion and dilatation, and arteriovenous malformations), which becomes a good detection method for diagnosing small intestinal telangiectasia with bleeding [16].

The treatment methods of small intestinal telangiectasia mainly include drugs, endoscopy and surgery [17,18]. However, since the small intestinal telangiectasia is limited to the submucosa, there is a higher probability of rebleeding after drug and endoscopic treatment, while the surgical treatment can be comprehensively checked to completely stop bleeding at the bleeding site, reduce the chance of rebleeding. In this report, as the patient underwent CT, gastroscopy and enteroscopy, and no abnormalities were found, However, through capsule endoscopy, blood clots were found in the middle and upper segment of jejunum. Through a comprehensive assessment, we decided to explore by laparotomy, the bleeding point in the jejunum was found finally, and then completely stopped the bleeding. We considered that this disease may have additional bleeding sites, therefore, we also conducted a comprehensive exploration of the whole intestine to ensure that no other bleeding points were missed. The patient’s condition was stable and recovered well after operation, and there was no recurrent gastrointestinal bleeding after follow-up. For the surgical treatment of small intestinal telangiectasia with bleeding, the key is to locate accurately and find the bleeding point during the operation, the entire intestine needs to be carefully inspected to prevent omission.

The following points should be noted during surgical treatment: (1). When the location of the lesion is unclear, can not rush to the surgical exploration, and corresponding examination should be carried out to initially determine the location of the bleeding; (2). Do not inflate too much during the operation of endoscopy to avoid compressing the bleeding and covering up the target bleeding point, and affecting the examination; (3). During the intraoperative examination, the operation is gentle to avoid causing intestinal edema; (4). For unclear positioning, large bowels should not be removed blindly; (5). This disease often has multiple site bleeding, the bleeding often stops on its own or after non-surgical treatment, and then recurs. Therefore, surgeons should carefully examine the intestines to avoid omissions; (6). The possibility of this disease should be considered in patients with negative results through some examinations such as gastroscopy, enteroscopy and gastrointestinal barium meal radiography.

## Funding

No funding.

## Ethical approval

Ethical approval has been exempted by our institution's ethics committee (The Ethics Committee of Nanchang University) as this publication is a case report, provided that patients/patient’s next-of-kin have given their informed written consent for the publication of this case report.

## Consent

An informed written consent was taken from the patient for reporting this case and the accompanying images.

## Author contribution

This report was performed by Wen-jun Zhang, some clinical information were provided by You-shan Huang, and was supported and guided by Zeng-ming Zhu and Hong-liang Luo.

## Registration of research studies

This is not first-in-human study, thus it is not needed.

## Guarantor

Wen-jun Zhang.

## Provenance and peer review

Not commissioned, externally peer reviewed.

## Declaration of Competing Interest

No conflict of interest in this paper.
